# Structural Characterization and In Vitro Antioxidant, Hypoglycemic and Hypolipemic Activities of a Natural Polysaccharide from Liupao Tea

**DOI:** 10.3390/foods12112226

**Published:** 2023-05-31

**Authors:** Lu Wei, Li Huang, Lijuan Du, Qinju Sun, Can Chen, Jie Tang, Jianwen Teng, Baoyao Wei

**Affiliations:** 1 College of Light Industry and Food Engineering, Guangxi University, Nanning 530004, China; weilu820301@163.com (L.W.); mary607@126.com (L.H.); 13640500549@163.com (C.C.); 18707757209@163.com (J.T.); tjw1027@gxu.edu.cn (J.T.); 2 Institute of Food and Pharmaceutical Science, Guangxi Vocational University of Agriculture, Nanning 530007, China; dlj2015bys@163.com (L.D.); sqj201211@163.com (Q.S.)

**Keywords:** Liupao tea polysaccharide, structure, biological activity, in vitro

## Abstract

This study extracted and purified a natural polysaccharide (TPS-5) that has a molecular weight of 48.289 kDa from Liupao tea, a typical dark tea with many benefits to human health. TPS-5 was characterized as a pectin-type acidic polysaccharide. It has a backbone composed of → 2,4)- α- L-Rhap-(1) → 4)- α- D-GalAp-(1) →, with a branch composed of → 5)- α- L-Ara-(1 → 5,3)- α- L-Ara-(1 → 3)- β- D-Gal-(1 → 3,6)- β- D-Galp-(1) →. The in vitro biological activity evaluation illustrated that TPS-5 has free radical scavenging, ferric-ion-reducing, digestive enzyme inhibitory, and bile-salt-binding abilities. These results suggest that TPS-5 from Liupao tea has potential applications in functional foods or medicinal products.

## 1. Introduction

Liupao tea, with a history of being drunk for more than 1500 years, is produced in Wuzhou, Guangxi, China [1]. The production process for Liupao tea can be briefly described as follows: fresh tea leaves are picked from local large-leaf tea trees, which go through fermentation, initial steaming, fermentation, re-steaming, compression, drying, natural aging, and other processes. Pile fermentation and natural aging are the key processes that form the singular savor of Liupao tea. In response to the hot and humid environment and microorganisms, polyphenols, carbohydrates, proteins, amino acids, and other ingredients in the raw materials of Liupao tea undergo microbial transformation, enzymatic oxidation, non-enzymatic automatic oxidation, degradation, condensation, and other complex chemical reactions, forming the special quality of Liupao tea. For example, Liupao tea soup is amber and reddish-brown in color; has the aroma of betel nut, bacterial flower or Chen Xiang; and tastes soft and smooth [2,3]. In 2022, the “Liupao Tea Making technique” was listed by UNESCO as an intangible cultural heritage of humanity. Li Shizhen of the Ming Dynasty in China wrote the Compendium of Materia Medica, in which he once mentioned that Liupao tea, especially aged tea, has the functions of cooling and relieving heat, warming the stomach, invigorating the spleen, helping digestion, and assisting in the treatment of heat stroke, cold, nausea and stomach pain. Many experimental studies have confirmed that Liupao tea has various biological functions, such as lowering blood sugar and lipids, inhibiting coagulation, improving intestinal flora, and having antioxidant properties [4,5,6].

The important active ingredient in tea is polysaccharide. Compared with other teas, polysaccharides in dark teas exhibit the strongest antioxidant power and inhibitory effects against α-glucosidase. Unlike green tea polysaccharides, dark tea polysaccharides are frequently transformed or metabolized by microorganisms from carbohydrates present in tea leaves [7]. They are mostly acidic heteropolysaccharides. Due to the special pile fermentation and aging processes, their structure is more complex and often bound to other components, such as polyphenols and proteins. Because of these reasons, their structure is relatively difficult to analyze [8,9].

Several studies have investigated the structure and function of Liupao tea polysaccharides. Wang et al. [10] investigated the connection between microbial colony structure and dominant carbohydrates. Their results confirmed that during the fermentation procedure, the increase in the water-soluble contents of pectins, polysaccharides, and carbohydrates was positively correlated with *Aspergillus*, *Blastobotrys*, *Staphylococcus*, and *Brachybacterium*, which could release extracellular enzymes or produce extracellular polysaccharides. Pang et al. [11] found that, after fermentation, hemicellulose content decreased and water-soluble pectin content increased significantly, which was significantly correlated with an increase in polysaccharide content. Qin et al. [1] demonstrated that aged Liupao tea contained more polysaccharides than raw tea. Compared to raw tea polysaccharides (RLTPS), aged tea polysaccharides (ALTPS) with a lower molecular weight showed higher thermal stability and asymmetry, as well as higher anticoagulant activity and bile-acid-binding ability. A higher molar ratio of Rha, Gal, and GalA was observed in refined ALTPS, which were all glycoprotein complexes containing the pyranose ring structure. Qiu et al. [12] extracted two polysaccharide components from Liupao tea, whose basic physical and chemical properties were different. During the simulated digestion process, polysaccharides combined with proteins and polyphenols were partially degraded and released polyphenols, while polysaccharides without proteins and polyphenols were not digested; however, both types of polysaccharides had the potential to improve gut flora. 

There is a close relationship between polysaccharides’ chemical structure and physiological activities, but few studies on the structure–activity relationship of Liupao tea polysaccharides have been reported. Current studies on the structure of Liupao tea polysaccharides are still limited to molecular weight, monosaccharide composition and proportion, and morphological characterization. It is crucial to research more structural information about Liupao tea polysaccharides, which is conducive to better explain the mechanism of this tea’s physiological function in order to make better use of it. Therefore, more structural details about Liupao tea polysaccharides, including the kinds of glycosidic bonds, as well as the sequence and location of monosaccharides, need to be further analyzed.

The aim of this study was to provide theoretical support for further analysis of the functional mechanism of Liupao tea polysaccharides. In this study, a polysaccharide called TPS-5 was isolated and purified from Liupao tea. Besides the basic information, such as functional group, molecular weight, and constituent monosaccharides and their proportions, chemical constituents were researched and a detailed analysis of the structural information of TPS-5 was performed, which included monosaccharide residue positions, connection mode, and sequence, as well as glycosidic bond configuration, among others. In addition, the free radical scavenging power, bile-salt-binding ability, ferric-ion-reducing property, and blood-glucose-reducing property of TPS-5 were evaluated in vitro. This study provided the first report on the detailed analysis of the structure of a purified polysaccharide from Liupao tea and preliminarily described the relationship between its structure and activity. The results provided a scientific basis for better understanding the physiological activity of Liupao tea polysaccharide and promoted its application in the food, cosmetic, healthy product, and pharmaceutical industries. According to the results, TPS-5 could be applied in foods, cosmetics, healthy products, and pharmaceuticals as a natural antioxidant, lipid-lowering, and glucose-lowering agent.

## 2. Materials and Methods

### 2.1. Materials and Reagents

Third-grade aged Liupao tea with No. 712030 was provided by China Wuzhou Tea Co., Ltd (Wuzhou, Guangxi, China).

Ethanol absolute, N-Butanol, trichloroacetic acid (TCA), and hydrogen peroxide solution (H_2_O_2_) were provided by Chengdu Kelon Chemical Co., Ltd. (Chengdu, China). 2,2-Diphenyl-1-picrylhydrazyl (DPPH), polyamide resin, 2,4,6-Tris(2-pyridyl)-s-triazine (TPTZ), carbazole, DEAE-52 cellulose, p-Nitrophenyl-β-D-Galactopyranoside (PNPG), acarbose, 3,5-Dinitrosalicylic acid (DNS), α-amylase, α-glucosidase, ferric chloride hexahydrate (FeCl₃·6H₂O), sodium acetate (NaAc), glucose (Glu), gallic acid (GalA) and bovine serum albumin (BSA) were supplied by Yuanye Biotech Co., Ltd. (Shanghai, China). Acetic acid was provided by Thermo Fisher Scientific (Shanghai, China). Sephadex G-200, sodium taurocholate, sodium glycocholate, Folin-phenol reagent, concentrated sulfuric acid, and ethyl acetate were provided by Solarbio Tech Co., Ltd. (Beijing, China). Sodium hydride, acetic anhydride, methyl iodide, and dimethyl sulfoxide (DMSO) were provided by Adamas-beta (Adamas, Shanghai, China). Deuterium oxide (D_2_O), acetone d6, trifluoroacetic acid (TFA), and ethylacetate were provided by McLean Biotech Co., Ltd. (Shanghai, China). Perchloric acid, sodium borohydride, methanol, dialysis bags, and dextran standard were provided by Sigma-Aldrich (St. Louis, MO, USA). Standard monosaccharides and methylation kits were supplied by Borui Biotech Co., Ltd. (Yangzhou, China).

### 2.2. Preparation Process of TPS-5

**Figure 1:**
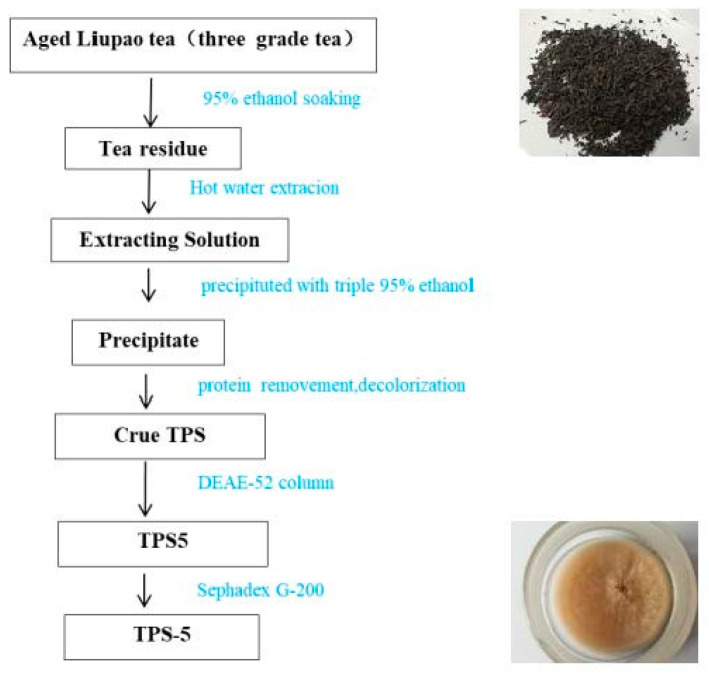
Procedure of TPS-5 extraction and purification from Liupao tea.

The preparation process was based on our previous method with slight modifications [4,12]. Figure 1 shows the preparation process of TPS-5. First, the tea was crushed and screened with an 80-mesh screen. In order to remove tea pigments, the tea powder was soaked in 95% ethanol for 18 h and filtered. Then, pure water was added to the tea powder at a ratio of 1:1 (*v*/*w*) and extracted at 90 °C for 2 h; the operation was repeated 3 times. The extraction solution was collected and concentrated, and then a triple volume of anhydrous ethanol was added to obtain tea polysaccharide precipitation. Next, the polysaccharide precipitates were redissolved in pure water, and proteins and tea pigments were removed via Sevag deproteinization and polyamide resin adsorption in sequence. After these operations, crude Liupao tea polysaccharides (Crude TPS) were obtained.

Then, the crude polysaccharides were purified using a DEAE-52 column (2.6 × 30 cm). In this procedure, 0, 0.1, 0.2, 0.3, 0.4, 0.5, and 1 m of NaCl was used successively as the elution solvent. The eluting flow rate was controlled at 15 mL/h, and each tube was collected for 20 min. The contents of uronic acid and carbohydrate in each tube were determined. With the number of elution tubes as the abscissa and the absorbance value as the ordinate, an elution curve was drawn. The elution fractions were concentrated, desalted, and lyophilized to obtain 6 polysaccharides, which were named TPS1, TPS2, TPS3, TPS4, TPS5, and TPS6, respectively. Sephadex G-200 gel column chromatography (1.75 × 70 cm) was used to purify TPS5 further. Pure water was used as the elution solution, and the elution, collection conditions, and elution curve drawing method were the same as those of ion-exchange chromatography. The symmetrical elution peaks were collected, and TPS-5 was obtained after lyophilization.

### 2.3. Structural Description of TPS-5

#### 2.3.1. Ultraviolet-Visible (UV) Spectrum Scanning

A total of 1 mg/mL of TPS-5 solution was scanned using a UV spectrophotometer (5500, Yuanxi, Shanghai, China) at 200–800 nm wavelengths [13].

#### 2.3.2. Molecular Weight (M_w_) Determination

The molecular weight of TPS-5 was measured using high-performance gel permeation chromatography (HPGPC) according to a previous method [14]. Using 0.05 M of NaCl solution as the eluent, 20 μL of 1 mg/mL of TPS-5 solution was injected into a superhydrogel linear BRT105-104-102 column (8 mm × 300 mm) in liquid chromatography (LC-10A, Shimadzu, Tokyo, Japan) with a differential detector (RI-10A, Shimadzu, Japan). The elution temperature was 38 °C, and the elution flow rate was 0.6 mL/min. The M_w_ of TPS-5 was determined using the standard curve of the T-series glucan standard.

#### 2.3.3. Monosaccharide Composition Analysis

This analysis was conducted in accordance with previous literature with some modifications [14]. TPS-5 was tested using ion chromatography with a CarboPac TMPA20 column (3 × 150 mm) and pulse-current sensor (IC-5000, Thermo Fisher, Milford, CT, USA). First, 5 mg of TPS-5 was hydrolyzed with 2 mol/L of TFA (10 mL) at 120 °C for 3 h. The TFA was completely blown away with nitrogen, and the samples were redissolved in deionized water; after centrifugation at 12,000 rpm/min, a 5 µL sample was injected into the ion chromatography instrument to detect monosaccharide composition. The mobile phase was A:H_2_O and B:250 mM NaOHC including 50 mM of NaOH and 500 mM of NaOAC; the flow rate was 0.3 mL/min; and the column temperature was 30 °C. The monosaccharide standards were used to identify and quantify the monosaccharides of TPS-5 by comparing the retention time and peak area.

#### 2.3.4. Fourier-Transform Infrared (FT-IR) Spectrum Analysis

According to the method in the literature [15], 250 mg of KBr and 2 mg of TPS-5 were mixed, extruded, and granulated. Then, the mixture was scanned using an FT-IR spectrometer (VERTEX 70 FT-_IR, Bruker, Germany) at the wavelength range of 400–4000 cm^−1^.

#### 2.3.5. Methylation Analysis

The methylation procedure was based on the literature with some modifications [8]. First, 50 mg of TPS-5 was reduced by 2 M of NaBH_4_ completely. Additionally, the solution was dialyzed (intercepted molecular weight of 4000 Da), and the holdup was lyophilized. Then, the reduced product was dissolved in 1.0 mL of anhydrous DMSO, followed by the addition of NaOH solution to the reaction system, and it was incubated for 12 h under dark conditions. Subsequently, 0.6 mL of methyl iodine was added in the dark for 5 h. The reaction mixture was then dilated for 48 h. After methylation, the product was hydrolyzed with 2 M of TFA at 120 °C for 2 h. Partially methylated alditol acetates (PMAA) were detected using gas chromatography-mass spectrometry (GC-MS-QP 2010 Ultra, Shimadzu, Japan) with an RXI-5 SIL MS column (Shimadzu, Japan) under the following temperature program: the injection port and detector temperature was 250 °C, the injection temperature was 120 °C, and the increasing rate was at 3 °C min^−1^, which was maintained at 250 °C for 5 min. The carrier gas was helium at a flow rate of 1 mL/min.

#### 2.3.6. Nuclear Magnetic Resonance (NMR) Spectrum Analysis

This analysis was performed according to the method reported in the literature [14]. First, 0.5 mL of D_2_O was added to 50 mg of TPS-5, and the sample was freeze-dried after full reaction at 4 °C for 24 h. Following that, the sample was redissolved in 0.5 mL of heavy water and analyzed using a 600 MHz NMR equipment (AVANCE III, Bruker, Billerica, MA, USA) performed at room temperature.

#### 2.3.7. Observation of Microscopic Morphology

According to our previous method [1], 5 mg of TPS-5 was pretreated via gold spraying and placed in an observation chamber. The micromorphology of TPS-5 was observed using a scanning electron microscope (SU8100 SEM, Hitachi, Tokyo, Japan).

### 2.4. Free Radical Scavenging and Ferric-Ion-Reducing Capacities of TPS-5

#### 2.4.1. DPPH Scavenging Test

This test was performed according to Li’s method with some modifications [14]. After mixing 1.00 mL of different concentrations (0.1, 0.2, 0.4, 0.6, 0.8, and 1.0 mg/mL) of the sample solution with 2.00 mL of 48 mg/L of DPPH solution, the reaction began for 30 min in the dark at 25 °C. The DPPH clearance rates were determined based on the following formula:

Clearance rate (%) = [1 − (As − Ac)/A_0_] × 100 (1)

where Ac and A_0_ represent the absorbance values at 517 nm of the samples without DPPH solution, the samples with DPPH solution, and pure water with DPPH solution, respectively.

#### 2.4.2. ABTS+ Scavenging Test

This test was based on Abuduwaili’s method with some modifications [16]. A total of 2.9 mL of 2.45 mmol/L of ABTS+ solution was blended with 0.1 mL of the sample solution at different concentrations (0.1, 0.2, 0.4, 0.6, 0.8, and 1.0 mg/mL) and reacted for 10 min at 30 °C. The calculation in Formula (1) was adopted in this test. As, Ac, and A_0_ represent the absorbance values at 734 nm of the samples without ABTS+, the samples with ABTS+, and the blank control, respectively.

#### 2.4.3. OH Scavenging Test

This test was based on Abuduwaili’s method with a few modifications [17]. A total of 2.0 mL of 9.0 mmol/L of FeSO_4_ solution, DNS solution, and H_2_O_2_ was added into 1.0 mL of TPS-5 solution at different concentrations (0.1, 0.2, 0.4, 0.6, 0.8, and 1.0 mg/mL) in turn. Then, the reaction began at 37 °C for 60 min. Formula (1) was used to calculate the clearance rate. As, Ac, and A_0_ represent the absorbance values at 510 nm of the samples without H_2_O_2_, the samples with H_2_O_2_, and the blank control, respectively.

#### 2.4.4. Ferric Iron Reducing Antioxidant Power (FRAP) Test

This test was based on Li’s method with some modifications [18]. The mixture of 150 μL of TPS-5 solution at different concentrations (0.1 to 1.0 mg/mL) with 4.5 mL of FRAP solution (300 mmol/L; pH of 3.6; NaAc solution, 10 mmol/L of TPTZ solution, and 20 mmol/L of FeCl_3_·6H_2_O at a volume ratio of 10:1:1) was reacted at 37 °C for 10 min. The FeSO_4_ solution with a series gradient of concentrations (0, 0.2, 0.4, 0.6, 0.8, and 1.0 mg/mL) was carried out in the above reaction under the same conditions. With the FeSO_4_ concentration value and the absorbance value at 593 nm as the horizontal and vertical coordinates, respectively, a standard curve was drawn to calculate the FeSO_4_ values, which represented activity. The ferric-reducing capacity of the sample was calculated as follows [19]:

Ferric-reducing power (mmol/L FeSO_4_) = Sample ferric-reduction power − Blank ferric-reduction power (2)

### 2.5. Bile-Salt-Binding Action

This analysis was based on the approach proposed by Huang et al. [20]. First, gradient solutions of 0, 0.05, 0.10, 0.15, 0.20, and 0.30 mmol/L of sodium taurocholate and sodium glycocholate were prepared with pure water. Then, 2 mL of 60% H_2_SO_4_ was added to the same volume of the above solution and reacted at 70 °C for 20 min. A standard curve was drawn with bile salt concentration and absorbance at 387 nm as the horizontal and vertical coordinates, respectively.

Then, 2 mL of 2.0 mg/mL of TPS-5 solution was blended with 2 mL of 0.01 mol/L of HCl solution and 2 mL of 10 mg/mL of pepsin solution, and the reaction was oscillated at 37 °C for 1 h (simulated gastric digestion environment). The pH was adjusted to 6.3 and 2 mL of 10 mg/mL of trypsin solution was added. The reaction was run for 1 h at 37 °C (simulated intestinal environment). Simulated gastrointestinal digestion samples (SGDS) of TPS-5 were obtained.

Next, 2 mL of SGDS solution was blended with 0.4 mmol/L of cholate solution of the same volume, and the reaction began at 37 °C for 1 h. Then, 2 mL of the supernatant was blended with 4 mL of 60% H_2_SO_4_ and continued to react at 70 °C for 20 min. By substituting the absorbance at 387 nm into the standard curve, the amount of residual bile salts in the reaction solution was calculated. The positive control was cholestyramine at a concentration of 1 mg/mL. Using Formula (3), the binding rate was determined as follows:

Bile-salt-binding rate (%) = (C_0_ − C_1_)/C_0_ × 100 (3)

where C_0_ represents the amount of bile salts, in μmol; and C_1_ represents the amount of residual bile salts, in μmol.

### 2.6. Digestive Enzyme Inhibition In Vitro

#### 2.6.1. Inhibition of α-Amylase

At 37 °C, 250 μL of α-amylase solution (1 U/mL) was mixed with 250 μL of sample solutions at different concentrations (0.1–5.0 mg/mL), and 500 μL of 1% starch solution (*w*/*v*) was then added. After 10 min, 1 mL of DNS reagent was added. The absorbance at 540 nm was determined after reaction in a 100 °C water bath for 5 min. The inhibition rate of α-amylase was calculated using the following formula [21]:

Inhibition rate (%) = 1 − (As − A_1_)/(A_b_ − A_2_) × 100 (4)

As and A_b_ represent the absorbance of amylase solution and phosphoric acid buffer and the absorbance of the sample solution and amylase solution, respectively. 

A_1_ and A_2_ represent the absorbance of phosphoric acid buffer and the absorbance of the sample solution and phosphoric acid buffer, respectively.

#### 2.6.2. Inhibition of α-Glucosidase 

A total of 50 μL of the sample solution at different concentrations (0.1–5.0 mg/mL), 100 μL of 0.7 U/mL of α-glucoside solution, 100 μL of 1 mM of PBS (pH 6.9) and 50 μL of 0.35 U/mL of PNPG solution were blended and reacted at 37 °C for 20 min. The reaction was terminated by adding 100 μL of 1 M of Na_2_CO_3_. The absorbance measured at 405 nm was substituted into the formula below [21]:

Inhibition rate (%) = [(A_0_ − As)/A_0_] × 100 (5)

A_0_ represents the absorbance of the blank control group, while As represents the absorbance of the sample group.

The carbose solution (0 to 5 mg/mL) was selected as the positive control for the amylase inhibition tests.

### 2.7. Spectrophotometric Analysis of Total Sugar, Protein, Total Polyphenol, and Uronic Acid Contents

This analysis was conducted according to our previous determination methods [12]. The content of total sugar was determined using the phenol–sulfuric acid method with glucose as the standard. First, 0, 0.2, 0.4, 0.6, 0.8, and 1.0 mL of standard glucose working liquid (0.1 mg/mL) was added in the test tube successively, pure water was then added up to 1.0 mL, and, finally, 5 mL of concentrated sulfuric acid was added to start the reaction. After 10 min, the absorbance at 490 nm was measured. A standard curve was drawn with the glucose content as the horizontal coordinate and the absorbance value as the vertical coordinate. A total of 1.0 mL of TPS-5 (0.5 mg/mL) solution was operated according to the above procedure, and the absorbance value at 490 nm was measured and then substituted into the standard curve to calculate the total sugar content.

The content of total polyphenol was determined based on the Folin-phenol method with gallic acid as the standard. First, 0, 0.1, 0.2, 0.3, 0.4, and 0.5 mL of gallic acid working liquid (0.1 mg/mL) was added in the test tube successively, pure water was then added up to 0.5 mL, and, finally, 5.0 mL of Folin-phenol solution (1 mol/L) was added. After 5 min, 4.0 mL of sodium carbonate solution (7.5%) was added to start the reaction. After the reaction was run for 1 h, the absorbance at 765 nm was measured. A standard curve was drawn with the gallic acid content as the horizontal coordinate and the absorbance value as the vertical coordinate. A total of 1.0 mL of TPS-5 (1.0 mg/mL) solution was operated according to the above procedure, and the absorbance value at 765 nm was measured and then substituted into the standard curve to calculate the total polyphenol content.

The content of protein was evaluated using the Bradford method with bovine serum albumin (BSA) as the standard. First, 0, 0.1, 0.48, 0.72, 0.84, and 0.96 mL of standard BSA working liquid (0.1 mg/mL) was added in the test tube successively, pure water was then added up to 1.0 mL, and, finally, 5 mL of G-250 (0.1 mg/mL) was added to start the reaction. After 5 min, the absorbance at 595 nm was measured. A standard curve was drawn with the standard protein content as the horizontal coordinate and the absorbance value as the vertical coordinate. A total of 1.0mL of TPS-5 (1 mg/mL) solution was operated according to the above procedure, and the absorbance value at 595 nm was measured and then substituted into the standard curve to calculate the protein content.

The uronic acid content was evaluated based on the carbazole–sulfuric acid method with galacturonic acid (GalA) as the standard. First, 0.0, 0.1, 0.2, 0.3, 0.4, and 0.5 mL of standard GalA working liquid (0.1 mg/mL) was added in the test tube successively, pure water was then added up to 0.5 mL, and, finally, 3 mL of sodium tetraborate–sulfuric acid solution (0.5 mg/mL) was added to start the reaction. After boiling in a water bath for 5 min, 0.1 mL of carbazole solution (1 mg/mL) was added, and the reaction continued in the boiling water bath for 5 min; the absorbance at 595 nm was measured. A standard curve was drawn with the standard GalA content as the horizontal coordinate and the absorbance value as the vertical coordinate. A total of 1.0 mL of TPS-5 (0.5 mg/mL) solution was operated according to the above procedure, and the absorbance value at 530 nm was measured and then substituted into the standard curve to calculate the uronic acid content.

### 2.8. Statistical Analysis

Three replications of each experiment were performed, and the results were shown as mean + SD. SPSS 17.0 was used for non-parametric analysis. The significance level was set as *p* < 0.05 to indicate significant difference. Origin 8.5 was used for plotting.

## 3. Results and Discussion

### 3.1. Structural Analysis of TPS-5

#### 3.1.1. Basic Structural Information of TPS-5

**Figure 2:**
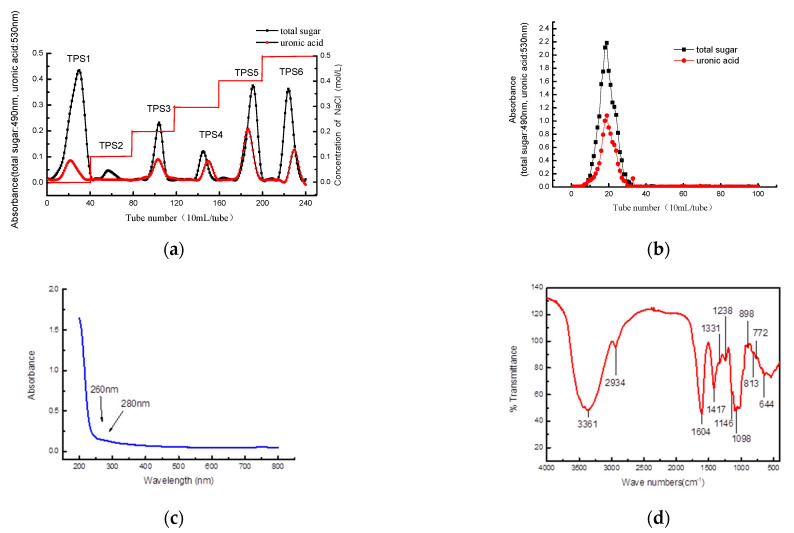
(**a**) DEAE-52 elution curve of crude Liupao tea polysaccharide; (**b**) Sephadex G-200 elution curve of TPS5; (**c**) UV absorption image of TPS-5; (**d**) FT-IR spectrum image of TPS−5.

After the separation of Liupao tea polysaccharides using the DEAE-52 (Figure 2a) and Sephadex G-200 column (Figure 2b), TPS-5 was obtained. The main chemical components of TPS-5 were as follows: the total sugar content was 53.32 ± 3.13%, the uronic acid content was 35.31 ± 2.23%, the protein content was 0.48 ± 0.23%, and the polyphenol content was 0.83 ± 0.47%.

The absorption peaks of TPS-5 at 260 nm and 280 nm are almost absent based on the UV spectrum scanning, which indicate that TPS-5 does not contain nucleic acid and protein (Figure 2c).

An analysis of the FT-IR spectrum of TPS-5 was performed, and the results are as follows: The wide and strong peaks at 3361 cm^−1^ are in response to the -OH vibration or stretch, while the absorption peaks at 2934 cm^−1^ are in response to the stretch of C-H [22]. An intense absorption peak at 1604 cm^−1^ indicates that TPS-5 contains -COOH [23], while the typical absorption peaks near 1098 cm^−1^ are in response to C-O-H and C-O-C pyranosaccharide rings [24]. Near 898 cm^−1^, there are peaks that indicate α-type glycosidic bonds, whereas the peak at 813 cm^−1^ indicates β-type glycosidic bonds [18,25] (Figure 2d). The results of FT-IR spectrum scanning indicate that TPS-5 is a kind of acidic polysaccharide with a complex structure.

#### 3.1.2. M_W_ and Monosaccharide Composition of TPS-5

**Figure 3:**
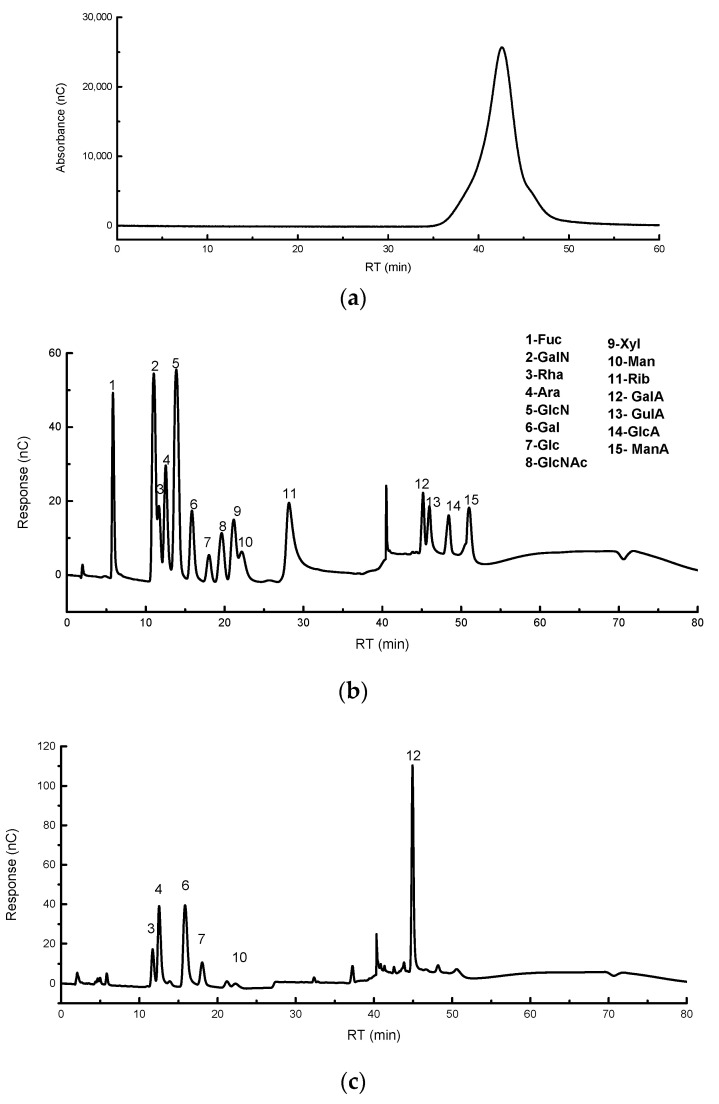
(**a**) High-performance liquid chromatography of TPS-5; (**b**) ion chromatograms of standard monosaccharides; (**c**) ion chromatogram of TPS−5.

The M_W_ was calculated to be 48.289 kDa based on the retention time of the dextran standard and the calibration curve combined with the retention time of TPS-5 (Figure 3a).

The monosaccharide composition analysis illustrates that TPS-5 has the characteristics of an acidic heteropolysaccharide. GalA, Gal, Ara, Rha, Glu, and Man are the main monosaccharide composition of TPS-5, and their molar ratio is about 0.398:0.191:0.120:0.077:0.143:0.023 (Figure 3b,c). Previous studies had found that there was a close relationship between the molar ratio of Gal, Man, Ara, and Xyl and the physiological function of polysaccharides [26]. Low M_W_ or high uronic acid polysaccharides had been found to usually have significant antioxidant activity, as well as hypoglycemic and free radical scavenging characteristics [27]. The basic structural information of TPS-5 is similar to that of a purified Fuzhuan tea polysaccharide called DTPS-6, which has been proven to have hypolipidemic and antioxidant properties [8].

In addition, the structural characteristics of TPS-5 are similar to ALTPS5, which was extracted from aged Liupao tea in our previous study. ALTPS5 has a lower M_W_ of about 30 kDa and a higher galacturonic acid content of about 54.95 ± 2.04%, as well as anticoagulant activity and bile-acid-binding capacity [1]. Combined with the information from the above analysis, TPS-5 could be preliminarily identified as an acidic pectin polysaccharide. More detailed information about the structural characterization and activities of TPS-5 is needed for further study.

#### 3.1.3. Methylation Analysis of TPS-5

GalA and its methylated form in TPS-5 were first reduced to the corresponding Gal residues before methylation analysis [28]. The methylation analysis results of polysaccharide methylated sugar alcohol acetyl ester (PMAA) are summarized in Table 1. Among them, GalAp-1-4 (50.24%) has the largest reduction and the isomerization form of Glcp-1-4, followed by GalAp-1-3-4 (14.36%). They are presumed to be the main components of the backbone of TPS-5. Araf-1-5 (5.35%), Araf-1-3-5 (2.69%), and Galp-1-3-6 (2.38%) are the next highest, which are speculated to form the branched-chain part of TPS-5. In addition, it was found that the ratio of Rhap-1-2-4 is also relatively high (5.17%); this finding suggests that TPS-5 may have the structural characteristics of rhamnogalacturonan [29,30]. These results also show that TPS-5 has both linear units and branched units, and the number of branched chains is large. According to Hawker’s calculation formula [31], the branching degree (DB) of TPS-5 was calculated to be 32.36%. According to the results of the methylation analysis, TPS-5 is a kind of pectin polysaccharide with GalA and Rha as the main chains and Arabinogalactan-II and galactan as the branched chains.

#### 3.1.4. NMR Analysis of TPS-5

According to the references in the literature [32,33,34,35] and NMR analysis results, the C/H chemical shift of TPS-5 is summarized in Table 2.

**Figure 4:**
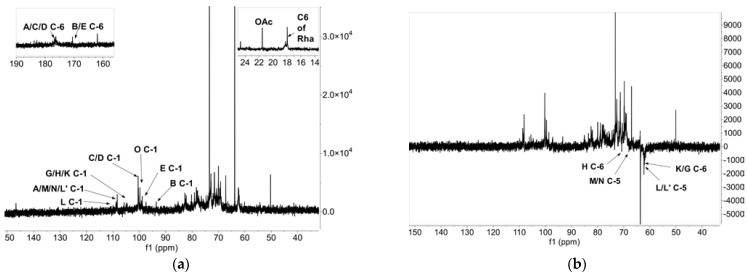

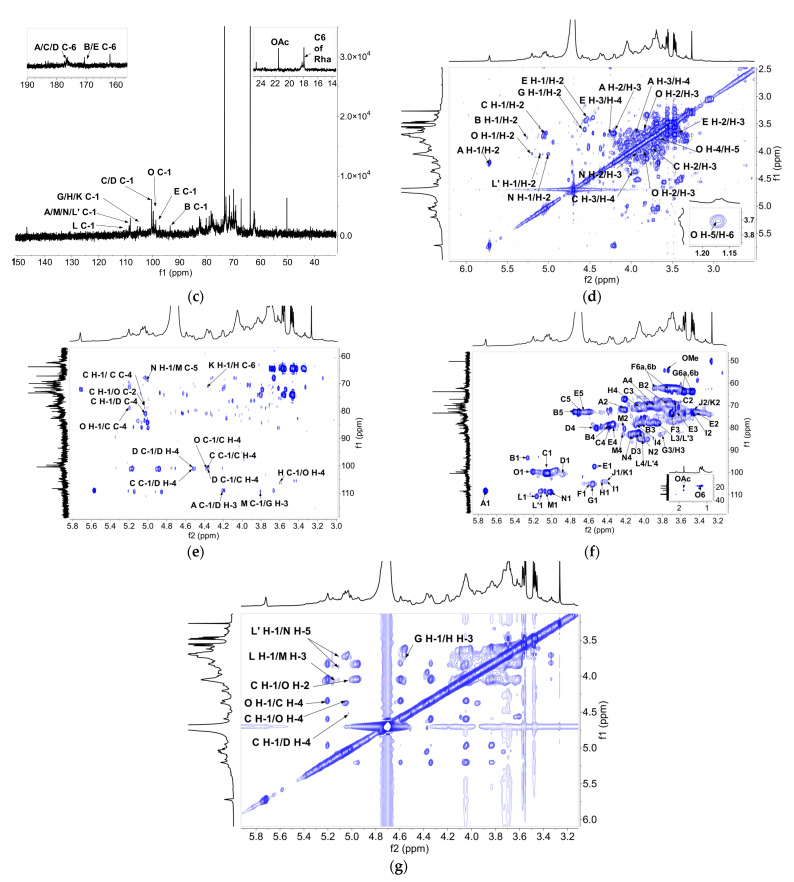
One-dimensional NMR spectroscopy of TPS-5: (**a**) ^13^C, (**b**) DEPT135, and (**c**) ^1^H NMR spectrum. Two-dimensional NMR spectroscopy of TPS-5: (**d**) COSY, (**e**) HSQC, (**f**) HMBC, and (**g**) NOESY spectrum.

The nuclear magnetic resonance carbon spectrum (^13^C NMR spectrum) (Figure 4a) shows that δ108.19 ppm, δ93.63 ppm, δ100.39 ppm, δ97.49 ppm, δ105.39 ppm, δ110.63 ppm, δ108.91 ppm, and δ99.81 ppm are ascribed to the heterocephalic carbon (C-1) of Residues A, B, C, E, G, L, M, and O. The signal at δ170.80 ppm is classified as C-6 of B and E, while the signals at δ176.51 ppm and δ176.71 ppm are classified as C-6 of A, C, and D.

In the DEPT135 spectrum (Figure 4b), the inverted signals at δ63.87 ppm, δ70.76 ppm, δ62.41 ppm, δ62.5 ppm, δ67.81 ppm, and δ68.28 ppm could be attributed to C5/C6 of G, H, K, L, M, and N, while the δ70.76 ppm, δ67.81 ppm, and δ68.28 ppm signals indicate that C5/C6 migrates to the lower field and there is substitution.

In the NMR hydrogen spectrum (^1^H NMR) (Figure 4c), δ5.71 ppm, δ5.04 ppm, δ4.84 ppm, 4.55 ppm, δ5.16 ppm, δ5.05 ppm, and δ5.21 ppm are the signals of the heterocephalic hydrogen (H-1) of Residues A, C, D, G, L, M, and O. Furthermore, δ1.19 ppm is the H-6 signal of Rha; δ1.81 ppm is the methyl H signal of acetyl group; and δ3.73 ppm is the signal of methoxy H [15].

Residue C (→4-α-D-GalpA-1→) was chosen as an illustration to analyze the other signals in 2D NMR. In the COSY mass spectrometry (Figure 4d), the signals at δ5.04 ppm, δ3.68 ppm, δ3.94 ppm, δ4.33 ppm, and δ4.69 ppm are from Residue C of H-1, H-2, H-3, H-4, and H-5, respectively. The cross peaks at δ5.04/3.68 ppm, δ3.68/3.94 ppm, δ3.94/4.33 ppm, and δ4.33/4.69 ppm are also detected based on the 1HNMR distribution results of the COSY spectrum. The carbon signals of Residue C were assigned based on the HSQC spectrum (Figure 4e). The H-1/C-1, H-2/C-2, H-3/C-3, H-4/C-4, and H-5/C-5 of Residue C cause the cross peaks at δ5.04/100.39 ppm, δ3.68/69.41 ppm, δ3.94/70.06 ppm, δ4.33/79.16 ppm, and δ4.69/72.66 ppm in the HSQC spectrum, while δ176.71 ppm is the signal of carboxyl carbon of Residue C. The cross peak at δ 54.23/3.73 ppm is the signal of the methyl group of C on O-6 [15,36]. 

The HMBC (Figure 4f) and NOESY (Figure 4g) spectra were used to confirm the residue sequences and binding sites of TPS-5. The glycosyl bonds between residues were determined based on the HMBC spectrum. The H-1 of Residue C shows a cross-signal peak (C H-1/C C-4) with its own C-4. The C-1 of Residue C shows a cross-signal peak (C C-1/C H-4) with its own H-4. These signals explain that there is a link within Residue C itself (C → C). The same method was used to analyze the connections of other glycosidic bonds. The cross-signal peaks of C H-1/D C-4 and C C-1/D H-4 show the presence of a connection between Residue C and Residue D (C → D), and the connecting site is located at the C-4 position. A strong correlation signal of D C-1/D H-4 indicates the existence of a D → D connection. The signal peaks of C H-1/O H-2 and C H-1/O C-2 indicate the existence of a C → O connection, while the cross-signal peaks of O C-1/C H-4 and O H-1/C C-4 in the HMBC spectrum and O H-1/C H-4 in the NOESY spectrum indicate the existence of O → C. Above all, the backbone of TPS-5 could be deduced to consist of a C → C → D → D → C → O → C linkage. This is evidenced by the cross peaks of A C-1/D H-3 that appear in the HMBC spectrum. It could be estimated that O-3 is the position where Residue A is linked to Residue D.

The HMBC and NOESY spectra were further analyzed using the same method as above. The cross signals of L’H-1/N H-5, N H-1/M C-5, M C-1/G H-3, G H-1/H H-3, as well as L H-1/M H-3, K H-1/H C-6 and H C-1/O H-4, appear. In summary, the branch of TPS-5 was deduced to consist of Residues L → N → M → G → H → [37]. Other glycosidic bonds related to Man and Glc could not be effectively identified using the NMR spectra due to their extremely weak signals. Man and Glc are likely to exist in the branched-chain portion at O-4 of Residues 1, 2, and 4-Rha as a result of TPS-5′s pectin characteristics. 

Based on the above analysis, we could infer the backbone of TPS-5 to be composed of →2,4)-α-L-Rhap-(1→4)-α-D-GalAp-(1→, while its branches are composed of →5)-α-L-Araf-(1→5,3)-α-L-Araf-(1→3)-β-D-Galp-(1→3,6)-β-D-Galp-(1→. Branches are linked to the backbone by the O-4 bond of the residue →2,4)-α-L-Rhap-(1→, which has also been reported by Tang [29].

**Figure 5:**
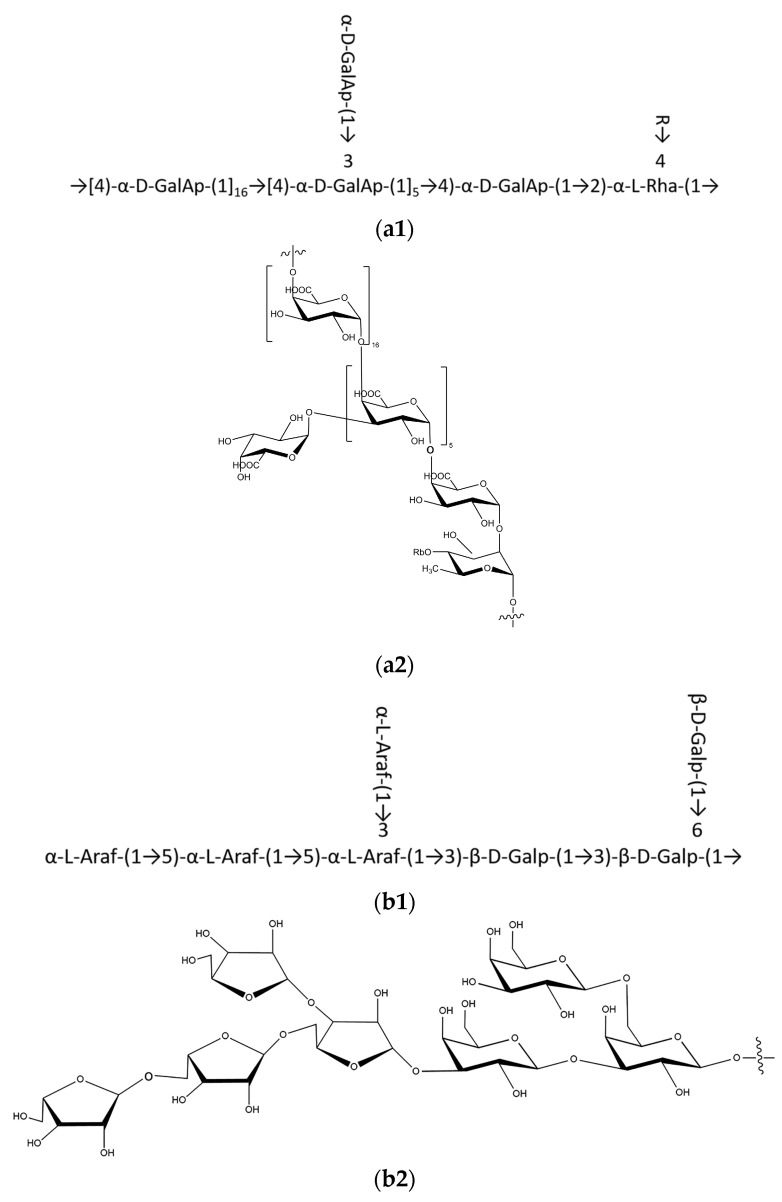
Deduced chemical structure of the repeating units of TPS-5: (**a1**,**a2**) backbone-repeating units and (**b1**,**b2**) branch-repeating units.

TPS-5 has typical pectin structural characteristics, and the structural formula is shown in Figure 5. The structure of a polysaccharide named DTP-1, which was extracted from Fu Brick tea, another famous dark tea in China, was analyzed by Liu et al. [38]. The results show that DTP-1′s structure is different from that of TPS-5. Its main chain mainly consists of Glc, Ara, and Galn, while its side chain consists of 4,6) -β-d-Galp-(1→. These differences are associated with their origin, raw materials, fermentation environment, and microorganisms. Fermentation and post-fermentation processes are the key steps in the manufacturing sequences of dark tea. During these processes, the composition of microorganisms has a very important influence on the formation of pectin. Pectin can make aged Liupao tea show different physiological activity from raw tea and form a mellow and rich taste [7,10,12]. Pectin has been proven to prevent hyperlipidemia, influence intestinal movement regulation, and have antioxidant and hypoglycemic activities. Pectin has been proven to prevent hyperlipidemia by lowering cholesterol, and it has also been proven to regulate intestinal movement, have antioxidant activity, and participate in glucose absorption [39,40]. Further research about the functional activity of TPS-5 is needed.

#### 3.1.5. SEM Analysis of TPS-5

**Figure 6:**
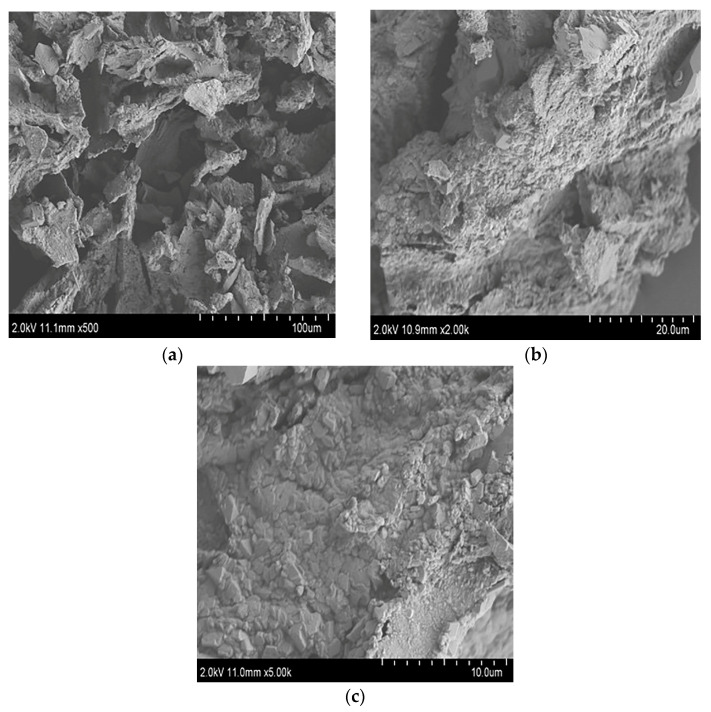
(**a**) SEM image of TPS-5 at 500×; (**b**) SEM image at 2000×; (**c**) SEM image at 5000×.

Under a 500-fold microscope, TPS-5 presents a granular and blocky cluster structure, which appears to have massive cavities inside it (Figure 6a); thus, it could possess preferable adsorption [41]. At 2000-fold, it could be seen to have a rough surface which looks like a dry and cracked soil layer (Figure 6b). At 5000-fold, it could be seen to have a surface with granular aggregates that are closely stacked together (Figure 6c). These phenomena may be the result of the strong intermolecular interaction of polysaccharides [15].

### 3.2. Biological Activity of TPS-5 In Vitro

#### 3.2.1. Evaluation of Free Radical Scavenging and Ferric-ion-Reducing Abilities of TPS-5

**Figure 7:**
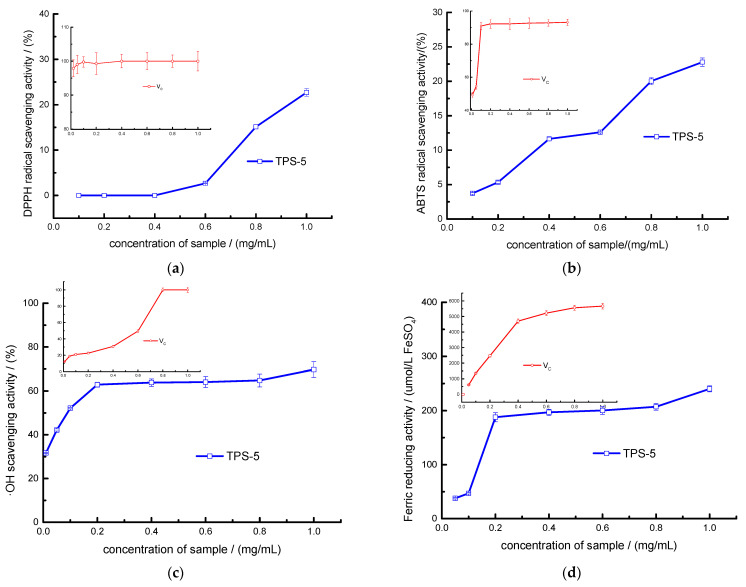
Antioxidant properties of TPS-5: scavenging power of (**a**) DPPH, (**b**) ABTS+, (**c**) OH, and (**d**) ferric-ion-reducing capacity.

The IC_50_ values of TPS-5 and Vc were 1.169 and 0.006 mg/mL for DPPH, 1.559 and 0.014 mg/mL for ABTS+, and 0.169 and 0.44 mg/mL for OH, respectively (Figure 7a–c). There was a positive correlation between TPS-5 concentrations and the scavenging effects of these free radicals at between 0.10 and 1.0 mg/mL. While in the range of 0.05–0.2 mg/mL, the ferric-ion-reducing capacity of TPS-5 also appeared to have a dose–effect relationship. At 1.0 mg/mL, the FRAP value of TPS-5 reached the maximum of 187.21 umol/L (Figure 7d). These research results illustrate that TPS-5 has a comprehensive antioxidant function in vitro. The DPPH and ABTS+ scavenging properties and ferric-ion-reducing activity of TPS-5 were weaker than V_C_, while its OH scavenging property was stronger than V_C_. The differences in antioxidant functions between these samples were due to their different physical and chemical properties, such as chemical compositions, molecular structures, and different antioxidant activity mechanisms [42].

The structural characteristics of TPS-5 are closely related to its antioxidant activity. The structural analysis showed that TPS-5 is a kind of pectin polysaccharide, which contains a lot of uronic acid and has many electron-absorbing groups, such as -CO-, -COOH, and -OAc. All of these have beneficial effects on antioxidation [43,44,45]. The carboxyl and carbonyl groups in acidic polysaccharides serve as additional electron-withdrawing groups and can serve as excellent hydrogen donors and electron-transfer agents, thereby enhancing antioxidant activity [46]. According to An’s report [47], high concentrations of galactose, mannose, arabose, and rhamnose have beneficial effects on the antioxidant activity of polysaccharides. Wang et al. [25] also found that polysaccharides with higher levels of uronic acid have stronger antioxidant activity. In addition, the higher content of reductive terminal -OH in the TPS-5 structure could also allow it to accept more free radicals and show an antioxidant effect [19].

#### 3.2.2. Bile-Salt-Binding Capacity of TPS-5

Cholate is the precursor component of human cholesterol synthesis. An increase in cholesterol can cause hyperlipidemia. Therefore, inhibiting the absorption of cholate plays an important role in reducing blood lipids. Studies have found that cholate can be excreted from the body after combining with tea polysaccharides, thus achieving the effect of lowering blood lipids [42]. In this study, sodium taurocholate and sodium glycocholate, which are common in cholate, were selected to investigate the potential hypolipidemic effect of TPS-5. Based on the results, TPS-5 possesses a certain binding ability to bile salt, indicating that it has potential application value in lowering blood lipids. The binding rate of TPS-5 to sodium taurocholate (31.46%) was better than to sodium glycocholate (10.50%). The inhibition rate of cholestyramine on bile salts was recorded as 100%, and the relative binding rate of TPS-5 to sodium taurocholate and sodium glycocholate was calculated to be 12.07 and 43.10%, respectively (Table 3). The binding ability of TPS-5 to bile salts was lower than that of cholestyramine, which might be caused by the fact that TPS-5 is a negatively charged soluble pectin polysaccharide, which forms a certain electrostatic repulsion with the same negatively charged cholate [1,6]. Gao et al. [48] found that Laminaria Japonica polysaccharide fragments containing high galactose and a high-branched-chain structure, as well as porous and disordered organizational morphology, had a stronger binding ability to bile acids. Sun et al. [49] indicated that the polysaccharide fragments from *Passiflora edulis* peel with a porous structure, high viscosity, and high uronic acid content showed high bile-acid-binding ability. Similar results have been found in the studies of bamboo polysaccharides [50]. In addition, dietary fiber has been reported to have bile-acid-binding ability [51]. TPS-5 is a kind of pectin polysaccharide, which is a kind of soluble dietary fiber. It has certain viscosity in aqueous solution and has a porous and disordered microscopic morphology. These characteristics can provide more binding active sites for bile acids.

This study further confirmed that TPS-5 is an important lipid reducing component in Liupao tea. Combined with the structural and morphological analysis of TPS-5, its bile-acid-binding ability is closely related to its porous structure characteristics, as well as its rich uronic acid content and hydroxyl groups [41,52].

#### 3.2.3. Hypoglycemic Ability of TPS-5

α-amylase can increase blood sugar levels by hydrolyzing starch [53], whereas α-glucosidase can release glucose by hydrolyzing glucosidase, thereby increasing the absorption of glucose and leading to the rise of blood glucose [54]. They are key enzymes in controlling blood sugar level, and effective inhibition of their activity can effectively control blood sugar level, thus preventing diabetes [55].

**Figure 8:**
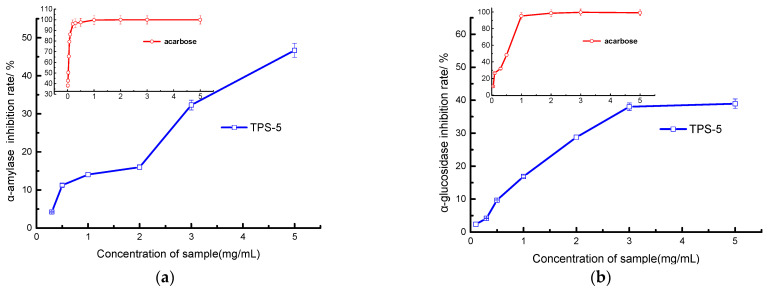
Hypoglycemic activities of TPS−5: (**a**) α-amylase and (**b**) α-glucosidase inhibition.

Figure 8 illustrates that the inhibitory capacity of TPS-5 toward the above two digestive enzymes has a dose–effect relationship. The IC_50_ values of TPS-5 and positive control acarbose are 4.923 and 0.035 mg/mL on α-amylase and 5.078 and 0.48 mg/mL on α-glucosidase, respectively. The clinical drug acarbose is a chemical synthetic drug, although it can effectively reduce postprandial blood sugar; however, if taken on a long-term basis, a variety of undesirable impacts will appear, such as diarrhea, bloating, skin allergies, and even liver damage [56]. TPS-5 is prepared from natural tea, which is natural, non-toxic, and safer than synthetic drugs.

The hypoglycemic activity of polysaccharides is closely related to their monosaccharide composition, glycosidic bond type, molecular weight, and spatial conformation [57]. The polysaccharide components in tea with hypoglycemic effects mostly belong to galactoglucan, with the 1→ 6) glucoside bond playing an important role in hypoglycemic activity [45]. The molecular weight of polysaccharides also has a great influence on their hypoglycemic activity, and the relationship between molecular weight and hypoglycemic activity is not simply positive or negative [1,12]. Both too-large and too-small molecular weights are not conducive to hypoglycemic activity [58]. Many studies have confirmed that plant polysaccharides have hypoglycemic effects [21]. For example, the IC_50_ values for α-amylase and α-glucosidase of Loquat leaf polysaccharides were found to be approximately in the range from 0.092 to 0.400 mg/mL and from 0.123 to 0.273 mg/mL, respectively [59]. Polysaccharides from red seaweed laver were observed to have an IC_50_ of 12.735 mg/mL on α-amylase [60], while polysaccharides extracted and purified from *Sargassum pallidum* had an IC_50_ between 0.071 to 4.344 mg/mL on α-glucosidase [61]. Thus, the hypoglycemic effects of these plant polysaccharides vary greatly. The differences in hypoglycemic efficacy of polysaccharides are closely related to the raw material source, extraction technology, structural characteristics, hypoglycemic mechanism, etc.

## 4. Conclusions

A natural polysaccharide, TPS-5, was extracted from Liupao tea, and its structure was analyzed. The monosaccharide composition and molecular weight detection results showed that TPS-5 is a kind of acidic polysaccharide with a pectin structure, and its M_W_ is 48.289 kDa. The detailed molecular structure of TPS-5 was elucidated in this study. The backbone of TPS-5 contains →2,4)-α-L-Rhap-(1→4)-α-D-GalAp-(1→, and the branches, which are linked to the O-4 position of →2,4)-β-L-Rhap-(1→, contain →5)-α-L-Araf-(1→5,3)-α-L-Araf-(1→3)-β-D-Galp-(1→3,6)-β-D-Galp-(1→. The results of the biological activity tests showed that TPS-5 has free radical scavenging, bile-salt-binding, hypolipemic, and hypoglycemic capacities and could be applied as a natural resource to regulate blood lipid or blood glucose function with antioxidant effects. These functions are closely related to the higher contents of uronic acid and hydroxyl groups, lower molecular weight, and porous morphological characteristics. This study provided new information on the structure–property–function relationship of a Liupao tea polysaccharide. In addition, it would be worthwhile to conduct further investigations on the structure of this Liupao tea polysaccharide and its biological activity mechanism in vivo.

## Figures and Tables

**Table 1:** Analysis of the TPS-5 linkage.

NO.	RT (min)	PMAA	Mass Fragments (*m*/*z*)	Area (%)	Linkage Type
1	10.358	2,3,5-Me3-Araf	43,71,87,101,117,129,145,161	0.71	Araf-1
2	15.475	2,3-Me2-Araf	43,87,99,101,117,129,189	5.35	Araf-1-5
3	18.483	2,3,4,6-Me4-Galp	43,71,87,101,117,129,145,161,205	2.88	Galp-1/GalAp-1
4	19.292	2-Me1-Araf	43,85,99,117,127,159,201,261	2.69	Araf-1-3-5
5	19.733	3-Me1-Rhap	43,87,101,117,129,143,159,189,203	5.17	Rhap-1-2-4
6	22.067	2,3,6-Me3-Galp	43,71,87,99,101,113,117,129,131,161,173,233	24.18	Galp-1-4/ GalAp-1-4
7	22.408	2,3,6-Me3-Glcp	43,71,87,99,101,113,117,129,131,161,173,233	26.06	Glcp-1-4/ GalAp-1-4
8	23.233	2,4,6-Me3-Galp	43,71,87,101,117,129,161,173,233	2.19	Galp-1-3
9	25.908	2,6-Me2-Galp	43,87,97,117,129,143,159,185	14.36	Galp-1-3-4/ GalAp-1-3-4
10	30.908	2,4-Me2-Galp	43,87,99,101,117,129,139,159,173,189,233	2.38	Galp-1-3-6

**Table 2:** The ^1^H, ^13^C NMR chemical shift data of TPS-5.

Glycosyl Residue	H1/C1	H2/C2	H3/C3	H4/C4	H5/C5	C6/H6a,b	Me/Ac
α-D-GalAp-(1→	5.71	4.20	3.65	3.88	ns		
(A)	108.19	72.11	71.32	70.71	ns	176.51	
→4)-α-D-GalAp	5.26	3.75	3.96	4.35	4.73		
(B)	93.63	69.61	75.31	79.13	72.71	170.80	
→4)-α-D-GalAp-(1→	5.03	3.68	3.94	4.33	4.69		3.73
(C)	100.39	69.41	70.06	79.16	72.66	176.71	54.23
→3,4)-α-D-GalAp-(1→	4.84	4.20	4.1	4.49	5.08		1.81
(D)	101.35	72.71	82.56	80.29	71.84	176.71	24.9
→4)-β-D-GalAp	4.52	3.42	3.67	4.28	4.67		
(E)	97.49	72.83	73.29	79.2	72.76	170.80	
→3)-β-D-Galp-(1→	4.55	3.58	3.80	4.12	3.67	3.56	3.48
(G)	105.39	72.1	83.22	69.74	76.9	63.87	
→3,6)-β-D-Galp-(1→	4.47	3.57	3.76	4.05	3.87	3.96	3.86
(H)	104.69	71.31	83.21	69.82	74.81	70.76	
β-D-Galp-(1→	4.36	3.45	3.58	3.86	3.71		
(K)	105.06	72.13	74.23	70.71	76.51	62.41	
α-L-Araf-(1→	5.16	4.12	3.89	4.07	3.73		
(L)	110.63	82.63	77.94	85.27	62.5		
α-L-Araf-(1→	5.12	4.06	3.88	3.97	3.72		
(L′)	108.63	83.61	78.14	85.24	62.51		
→3,5)-α-L-Araf-(1→	5.07	4.22	4.01	4.25	3.86,3.74		
(M)	108.91	80.62	83.66	83.31	67.81		
→5)-α-L-Araf-(1→	5.02	4.08	3.95	4.16	3.86,3.74		
(N)	108.89	82.19	78.13	83.69	68.28		
→2,4)-α-L-Rha-(1→	5.21	4.02	3.85	3.61	3.78	1.19	
(O)	99.81	78.76	75.11	76.81	74.76	17.96	

**Table 3:** Bile-salt-binding ability of TPS-5.

Treatment	Bile-Salt-Binding Ability (%)	
	Sodium Taurocholate	Sodium Glycocholate
TPS-5	10.50 ± 0.70a	31.46 ± 0.71a
Cholestyramine	86.98 ± 0.85b	73.00 ± 0.17b
TPS-5 relative to cholestyramine	12.07 ± 0.47	43.10 ± 0.25

In the same column, different letters indicate a significant difference at *p* < 0.05.

## Data Availability

The data presented in this study are available from the corresponding author upon request.
